# Impaired cerebral autoregulation detected in early prevasospasm period is associated with unfavorable outcome after spontaneous subarachnoid hemorrhage: an observational prospective pilot study

**DOI:** 10.1186/s13089-024-00371-8

**Published:** 2024-04-15

**Authors:** Edvinas Chaleckas, Vilma Putnynaite, Indre Lapinskiene, Aidanas Preiksaitis, Mindaugas Serpytis, Saulius Rocka, Laimonas Bartusis, Vytautas Petkus, Arminas Ragauskas

**Affiliations:** 1 Health Telematics Science Institute, Kaunas University of Technology, K. Barsausko str. 59, Kaunas, LT-51423 Lithuania; 2 Clinic of Anesthesiology and Intensive Care, Faculty of Medicine, Vilnius University, Vilnius, Lithuania; 3 Clinic of Neurology and Neurosurgery, Faculty of Medicine, Vilnius University, Vilnius, Lithuania; 4 Vilnius University Hospital Santaros Klinikos, Vilnius, Lithuania

**Keywords:** Subarachnoid hemorrhage, Delayed cerebral ischemia, Cerebral blood flow autoregulation, Baroreflex, Snapshot examination, Transcranial Doppler

## Abstract

**Background:**

Subarachnoid hemorrhage (SAH) patients with cerebral autoregulation (CA) impairment at an early post-SAH period are at high risk of unfavorable outcomes due to delayed cerebral ischemia (DCI) or other complications. Limited evidence exists for an association between early-stage CA impairments and SAH patient outcomes. The objective of this prospective study was to explore associations between CA impairments detected in early post-SAH snapshot examinations and patient outcomes.

**Methods:**

The pilot observational study included 29 SAH patients whose CA status was estimated 2–3 days after spontaneous aneurysm rupture and a control group of 15 healthy volunteers for comparison. Inflatable leg recovery boots (reboots.com, Germany) were used for the safe controlled generation of arterial blood pressure (ABP) changes necessary for reliable CA examination. At least 5 inflation‒deflation cycles of leg recovery boots with a 2–3 min period were used during examinations. CA status was assessed according to the delay time (∆T_CBFV_) measured between ABP(t) and cerebral blood flow velocity (CBFV(t)) signals during artificially induced ABP changes at boot deflation cycle. CBFV was measured in middle cerebral artery by using transcranial Doppler device.

**Results:**

Statistically significant differences in ∆T_CBFV_ were found between SAH patients with unfavorable outcomes (∆T_CBFV_ = 1.37 ± 1.23 s) and those with favorable outcomes (∆T_CBFV_ = 2.86 ± 0.99 s) (*p* < 0.001). Early assessment of baroreflex sensitivity (BRS) during the deflation cycle showed statistically significant differences between the DCI and non-DCI patient groups (*p* = 0.039).

**Conclusions:**

A relatively small delay of ∆T_CBFV_ <1.6 s between CBFV(t) and ABP(t) waves could be an early warning sign associated with unfavorable outcomes in SAH patients. The BRS during boot deflation can be used as a biomarker for the prediction of DCI.

**Trial registration:**

ClinicalTrials.gov Identifier: NCT06028906. Registered 31 August 2023 - Retrospectively registered, https://www.clinicaltrials.gov/study/NCT06028906.

## Background

Spontaneous aneurysmal subarachnoid hemorrhage (SAH) is a severe hemorrhagic stroke associated with the early onset of multiple complications that can lead to poor outcomes [1, 2]. The mortality rate after SAH is up to 35%, and more than one-third of survivors remain severely disabled [3].

After SAH, breakdown products of hemorrhaged blood release bioactive compounds that trigger inflammatory reactions leading to serious complications [4]. Cerebral vasospasm (CV) and delayed cerebral ischemia (DCI) are typical phenomena that occur in the human brain within 4–21 days after SAH [4, 5]. In addition, other complications, such as hydrocephalus, edema, sepsis and even rebleeding, may occur as a result of inflammation and disrupted cerebrospinal fluid circulation [3, 6, 7].

There is an urgent need to predict the clinical course in the first 1–3 days after SAH to mitigate possible complications. Different measures related to cerebral blood flow autoregulation (CA)— invasive pressure reactivity index (PRx), non-invasive TCD-based mean flow index (Mx) or phase shift (PS) between slow waves vasogenic B-waves [8] of arterial blood pressure (ABP) and cerebral blood flow velocity (CBFV), and baroreflex sensitivity (BRS) index—have been suggested as possible predictive factors that are considered feasible for personalized treatment in SAH patients.

Although several pilot studies have shown that CA impairment assessed in the first 1–4 days after SAH (early prevasospasm period) by PRx, Mx, and PS is associated with outcome after SAH [9–12], there is still limited evidence about the association of early CA impairment with DCI or CV.

Studies show that early deterioration of CA status is strongly associated with DCI in patients with large-artery CV after SAH [9, 11], but associations with vasospasm are contradictory in different studies [11, 13, 14]. Some studies show that CA impairment is observed before appearing the symptoms of CV [11, 14], therefore, the CA assessment in the early period would be useful for predicting cerebral vasospasms and outcome. However, other authors demonstrate that early TCD-based examination of CA status can be used to predict unfavorable outcome or cerebral infarction but not CV [10, 12, 13]. Monitoring optimal cerebral perfusion pressure (CPP) has been suggested to individualize CPP regulation-based management to avoid regional hypoperfusion in patients with SAH [9, 15]. It was demonstrated that optimal CPP increased approximately 30 h before the onset of DCI [16].

Invasive PRx monitoring is suggested for long-term continuous CA assessment and personalized SAH management in the intensive care unit (ICU). However, it requires invasive intracranial pressure (ICP) monitoring, which is not always appropriate for agitated or semiconscious patients with SAH, particularly in 4–14 days after SAH when the risk of CV occurrence is high. Noninvasive assessment of CA based on cerebral blood flow velocity monitoring by using transcranial Doppler (TCD) and calculation of the Mx or PS could be a rational solution for short CA monitoring sessions lasting up to a few hours [10, 11, 13, 17, 18] but not for long-term daily bedside monitoring.

The key limitation of Mx- and PRx-based monitoring approaches is that for reliable CA assessment, the presence of slow B-waves is necessary [19]. As slow B-waves are intermittent, the absence of slow waves can increase variation in measured CA indices, thus distorting diagnostic information [8, 20]. The variation in Mx and other CA indices might also be because different methodologies of assessing CA indices and simplified quantifications of a complex physiological mechanism were applied [20].

Mechanical generation of ABP changes or slow ABP waves was introduced to reduce the time required for CA assessment as well as to reduce the variation in CA indices. Repeated body position changes [21], sit-stand maneuvers [22], transient hyperemic response tests [12, 14, 23], deep breathing [24], pressure-cuff release tests [25–27] or noradrenaline infusion [28] are used to assess the dynamic response of cerebral blood flow and CA status. However, the application of most of these tests is limited in clinical practice due to strong interactions in critically ill neurosurgical patients.

To solve the aforementioned problem, we suggested the use of a commercially available pressotherapy device with inflatable boots capable of generating safe repeatable changes in ABP necessary for rapid CA assessment. The proposed method provides a much slower boot deflation cycle ($\sim$ 10 s) with a controlled ABP amplitude and therefore can overcome the disadvantages of the thigh cuff technique related to the risk of secondary brain injuries in critically ill patients.

The purpose of this observational clinical study was to explore the associations of CA impairments detected in the first 1–3 days after SAH with the clinical course of SAH patients by performing TCD-based snapshot CA examinations with ABP manipulations.

## Methods

### Design and setting of the study

The pilot observational study was conducted at Center for Anesthesiology, Intensive Care and Pain Management at Vilnius University Hospital Santaros Clinics (Lithuania), from 29 June 2021 to 30 June 2023. The study involved the assessment of CA status in 29 patients with spontaneous SAH (after aneurysm clipping surgery) and 15 healthy volunteers (control group) for comparison. Patients’ inclusion criteria: patients after SAH who received routine nursing in ICU after aneurysm clipping surgery. All patients were older than 18 years. Patients received routine care in accordance with the Lithuanian Guidelines on the Diagnosis and Management of Head Injury (2010) and the National Methodical Recommendations for the Diagnosis and Treatment of Brain Injury (2017).

### Ethical considerations

Clinical data collection was carried out in accordance with study protocols approved by the Vilnius Regional Biomedical Research Ethics Committees (Protocol No. 2021/6-1364-838). Participants and/or their legal guardians signed a written informed consent to participate in the study and use their anonymized clinical data for retrospective analysis. The study protocol was retrospectively registered at ClinicalTrials.gov Protocol Registration and Results System (Identifier: NCT06028906).

### Assessment of cerebral blood flow autoregulation

**Fig. 1:**
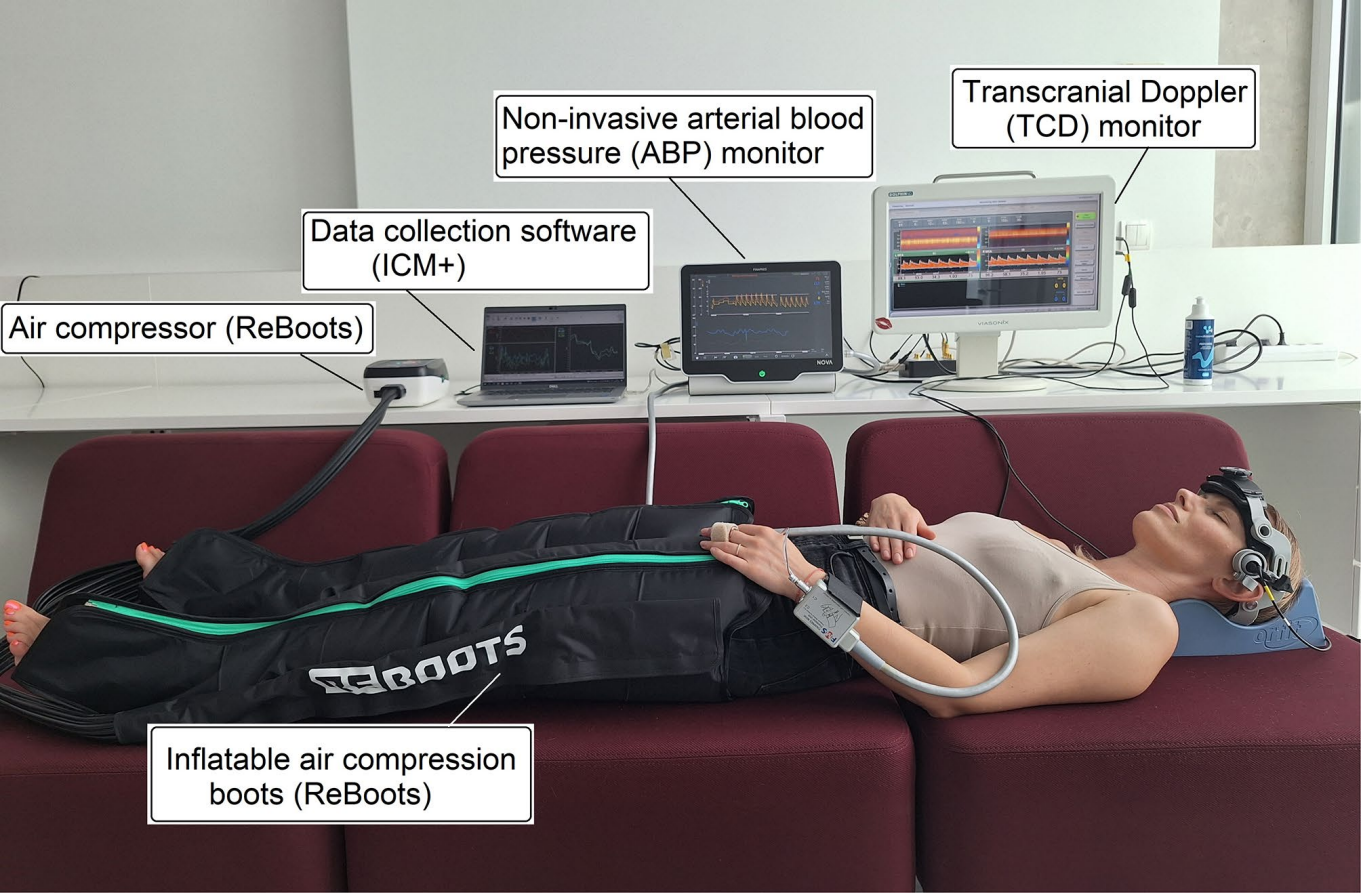
Generation of artificial ABP changes for assessment of CA in healthy volunteer by using air compression boots

Noninvasive CA status examination was performed in the early prevasospasm period: 2–3 days after aneurysm rupture, before the onset of CV or DCI. A pressotherapy device (leg recovery inflatable boots (Reboots, Germany) was used to generate artificial ABP changes required to assess CA dynamics (Fig. 1). The inflatable boots were placed on the patients’ legs (calf and thigh) to apply external pressure within safe controlled pressure limits not exceeding 200 mmHg during the maximal inflation phase. The boots were periodically inflated and deflated to generate artificial ABP changes: periodic slow ABP increase and fast 10 s drop during deflation by $\sim$ 10 mmHg with a 2–3 min period.

Multi-DopT (Compumedics DWL GmbH, Germany) and Dolphin 4D (Viasonix Ltd, Israel) TCD monitors were used to record transient responses of cerebral blood flow velocity (CBFV(t)) measured in the middle cerebral artery (MCA) in relation to induced ABP changes. The CA status was estimated by measuring the time delay between ABP(t) and CBFV(t) changes (∆T_CBFV_) during boots deflation cycle assuming that observed ∆T_CBFV_ values of 2…4 s reflect intact cerebral blood flow autoregulation, while lower values are associated with impaired CA [25, 26].

### Assessment of baroreflex sensitivity

The baroreflex sensitivity (BRS) was assessed together with CA examinations while performing ABP manipulation with inflatable boots. The BRS indices, calculated as a ratio of heart interbeat interval (∆T_IBI_) and ABP changes [29], were analyzed to explore their associations with later development of DCI. The methodology of applying BRS indices for outcome prediction after SAH was previously tested using naturally spontaneous ABP waves [30–33]. In our study, we calculated BRS indices during the boot deflation cycle at the maximum slope moment of ABP drop (BRS_drop_) and at the moment of ABP recovery after the nadir point (BRS_recovery_).

### Data collection

**Fig. 2:**
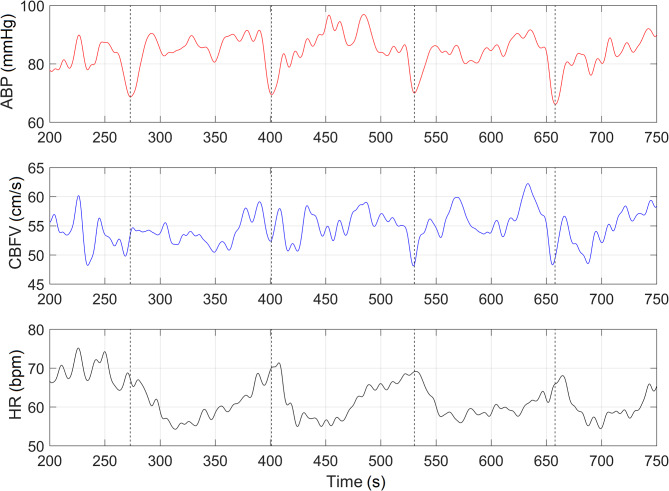
Example of ABP changes generated during deflation moments and registered responses in cerebral blood flow velocity (CBFV) and heart rate (HR). Dashed lines show the peak of ABP drop (nadir) moments during deflation cycle, which were used to synchronize responses from each boot deflation cycle in order to calculate average response of CBFV

At least 5 inflation‒deflation cycles were used to estimate the average transient response of ABP, CBFV and heart rate (HR) data for each participant. Recorded responses of ABP, CBFV and HR were synchronized according to ABP nadir moments from each deflation cycle to calculate average values of ∆TCBFV and BRS indices (Figs. 1 and 2). The total duration of the examination did not exceed 20 min.

In addition, the mean flow autoregulation index Mx, as a Pearson correlation coefficient between slow waves of ABP(t) and CBFV(t) within a 5-minute moving time window [34], was calculated during the examination sessions for comparison.

All monitoring data were collected using ICM + software (Cambridge Enterprise Ltd., UK). The sampling frequency of recorded ABP(t) and CBFV(t) data was 200 Hz. To eliminate instrumental delay, recorded CBFV(t) and ABP(t) data coincided in the time domain according to heartbeat variability. The CBFV(t) and ABP(t) data were filtered with a 5th order Butterworth low-pass filter with a cutoff frequency of 0.1 Hz and downsampled to 0.1 Hz before calculating the time delay ∆T_CBFV_.

### Patient outcome assessment

The Glasgow Outcome Score (GOS) was assessed 30 days after ictus or at hospital discharge, classifying patients into the following categories: deceased (GOS = 1), vegetative (GOS = 2), severe disability (GOS = 3), moderate disability (GOS = 4) and good recovery (GOS = 5). Computed tomography (CT) and computed tomographic angiography (CTA) scans were performed routinely to evaluate CV/DCI occurrence at 7th day after SAH or when clinical symptoms together with TCD examination showed incidences of CV (moderate CV when CBFV_MCA_ is 120–200 cm/s; critical CV when CBFV_MCA_ > 200 cm/s). Additionally, SAH patients were classified into groups according to whether they had been diagnosed with CV or DCI. For each study participant, CA-related parameters (∆T_CBFV_, Mx, BRS) were calculated and compared between the SAH patients and control groups. SAH patients were divided into outcome groups of fatal (GOS = 1) vs. survivors (GOS = 2…5) and unfavorable (GOS = 1…3) vs. favorable (GOS = 4…5).

### Statistical analysis

The Mann–Whitney U test or Fisher’s exact test were applied to estimate statistically significant differences in selected parameters between groups, which was indicated by *p* < 0.05. The threshold values of selected parameters that significantly separate outcome groups were identified according to the chi-square maximum value. The distribution of data among groups was expressed using either the mean and standard deviation (std) or the median and interquartile range (IQR). MatLab R2016a software was used for data processing and statistical analysis.

The Mann–Whitney U test (for ordinary variables) or Fisher’s exact test (for binary variables) were applied to estimate statistically significant differences in selected parameters between groups, which was indicated by *p* < 0.05.

The threshold values of parameters that significantly separate outcome groups were identified by forming series of 2 × 2 tables grouping the patients according to selected parameter into different outcome groups at different thresholds, and identifying the best discriminative threshold according to the highest chi-square value. The distribution of data among groups was expressed using either the mean and standard deviation (std) or the median and interquartile range (IQR). MatLab R2016a software was used for data processing and statistical analysis.

The sample size (29 SAH patients following aneurysm clipping surgery and 15 healthy volunteers) was impacted by project resource limitations, the constraints imposed by the pandemic and the limited study period (June 29, 2021 - June 30, 2023). Additionally, the modest sample size was influenced by the difficulties in obtaining high-quality signals during TCD examinations, by participant (or by their legal guardians) reluctance to participate in the study or their difficulties tolerating the pressure exerted by inflatable boots during the experiment. Accordingly, the non-parametric Mann–Whitney U test was chosen for its suitability with limited sample sizes and independence from the normal distribution of data, ensuring robust evaluation of statistically significant differences in selected parameters between groups.

## Results

SAH patients’ demographic data, clinical data and estimated CA-related metrics are presented in Tables 1 and 2, grouping patients according to outcome into survival vs. fatal outcomes (Table 1) and into favorable vs. unfavorable outcomes (Table 2). From the study population (29 SAH patients), 8 patients had fatal outcomes (28%), 23 patients had unfavorable outcomes (79%, including patients with fatal outcomes), and 6 patients had good recovery (21%). CV was detected in 10 patients (34%) in the later phase of the clinical course (4–9 days after SAH) according to computed tomography angiography (CTA) examinations or clinical symptoms. DCI was detected in 8 SAH patients (28%) according to computed tomography (CT) scans. All patients with DCI diagnosis experienced CV. No one had symptoms of CV during CA examination in the early period (2–3 days after SAH).

**Table 1:** Patient demographics and clinical examination data (fatal, survivors and healthy control groups)

	Fatal GOS 1	Survivors GOS > = 2	Healthy control	p (Fatal, Surv.)	p (Fatal, Cont.)	p (Surv., Cont.)
Number	8	21	15	-	-	-
Age, years median [IQR]	68 [58–74]	66 [54–75]	37 [33–40]	0.84	< 0.001*	< 0.001*
Sex, M/F	5/3	11/10	12/3	0.65	-	-
GCS, IQR	[5–6]	[8–12]	-	0.0034*	-	-
CV, n	3	7	-	0.46	-	-
DCI, n	1	7	-	0.28	-	-
∆T_CBFV_, s mean (std)	0.74 (0.56)	2.05 (1.35)	2.76 (0.72)	0.007*	< 0.001*	0.034*
Mx mean (std)	0.43 (0.14)	0.30 (0.24)	0.33 (0.08)	0.14 0.27	0.078 0.26	0.46 0.35
BRS_drop,_ ms/mmHg mean (std)	-0.25 (8.11)	-2.97 (8.66)	9.65 (6.86)	0.3	0.004*	< 0.001*
BRS _recovery,_ ms/mmHg mean (std)	-5.78 (5.29)	-7.91 (8.12)	-5.67 (4.94)	0.74	1	0.49

GOS is the Glasgow outcome scale, GCS is the Glasgow coma scale, CV is the cerebral vasospasm, DCI is the delayed cerebral ischemia, ∆T_CBFV_ is the time difference between cerebral blood flow velocity and arterial blood pressure, Mx is the mean flow autoregulation index, BRS_drop_ and BRS_recovery_ are the baroreflex sensitivity indices calculated as ratios between changes in interbeat interval and changes in arterial blood pressure (∆T_IBI_/∆ABP) during the boot deflation cycle at falling and recovery moments, IQR is the interquartile range, std is the standard deviation, Surv. is the survivor group, and Cont. is the control group. The asterisk (*) indicates statistical significance (*p* < 0.05)

**Table 2:** Patient demographics and clinical examination data (unfavorable, favorable outcomes and healthy control groups)

	Unfavorable GOS 1–3	Favorable GOS 4–5	Healthy control	p (Unfav., Fav.)	p (Unfav., Cont.)	p (Fav., Cont.)
Number	23	6	15	-		
median [IQR]	69 [61–77]	54 [49–55]	37 [33–40]	0.006*	< 0.001*	0.003*
Sex, M/F	10/13	6/0	12/3	0.016*		
GCS, IQR	[5–11]	[7–11]	-	0.34		
CV, n	9	1	-	0.33		
DCI, n	7	1	-	0.53		
∆T_CBFV_, s mean (std)	1.37 (1.23)	2.86 (0.99)	2.76 (0.72)	0.013*	< 0.001*	0.91
Mx mean (std)	0.36 (0.22)	0.23 (0.16)	0.33 (0.08)	0.15 0.088	0.55 0.72	0.17 0.056
BRS_drop,_ ms/mmHg mean (std)	-0.93 (8.20)	-6.83 (8.42)	9.65 (6.86)	0.17	< 0.001*	< 0.001*
BRS _recovery,_ ms/mmHg mean (std)	-7.89 (7.1)	-5.12 (8.74)	-5.67 (4.94)	0.25	0.39	0.61

GOS is the Glasgow outcome scale, GCS is the Glasgow coma scale, CV is the cerebral vasospasm, DCI is the delayed cerebral ischemia, ∆T_CBFV_ is the time difference between cerebral blood flow velocity and arterial blood pressure, Mx is the mean flow autoregulation index, BRS_drop_ and BRS_recovery_ are the baroreflex sensitivity indices calculated as ratios between changes in interbeat interval and changes in arterial blood pressure (∆T_IBI_/∆ABP) during the boot deflation cycle at falling and recovery moments, IQR is the interquartile range, std is the standard deviation, Unfav. is unfavorable outcome, Fav. is favorable outcome, and Cont. is the control group. The asterisk (*) indicates statistical significance (*p* < 0.05)

**Fig. 3:**
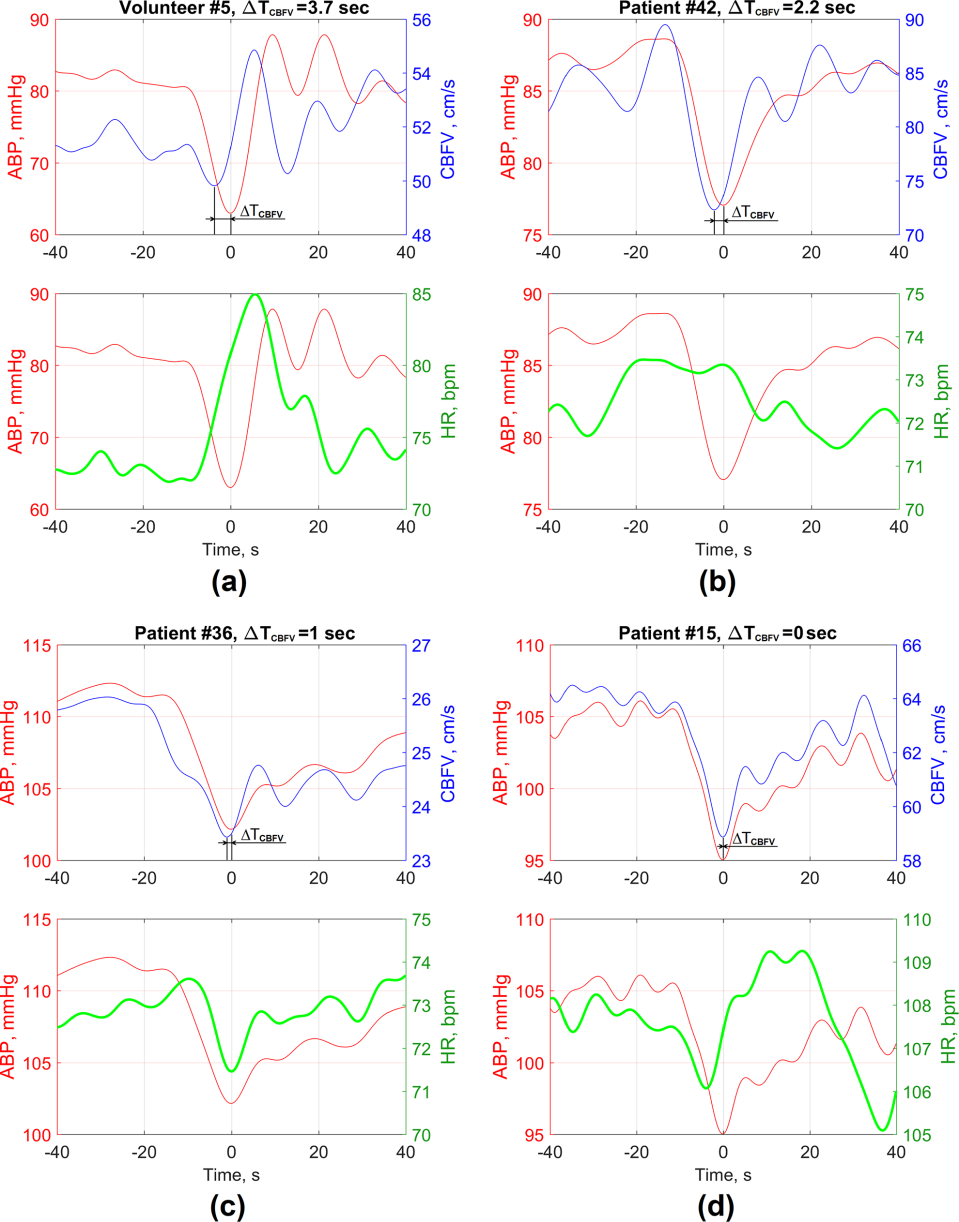
Examples of averaged transient responses of human cerebral blood flow autoregulation function in healthy volunteers (**a**) and SAH patients with favorable outcomes (**b**), severe disability with DCI (**c**) and fatal outcomes without DCI (**d**). The time moments at t = 0 show the peak of ABP drop (nadir) moments during the boot deflation cycle. The patients with later development of DCI are characterized by synchronous ABP and HR variation during the boot deflation phase (**c**)

Examples of transient responses of CBFV(t) and HR(t) caused by ABP(t) drop during the boot deflation cycle for healthy volunteers and SAH patients with favorable and unfavorable outcomes are shown in Fig. 3. Examples show that the CBFV(t) response overtook ABP(t) signal during deflation cycle due to active cerebral blood flow autoregulatory response [27], thus causing 2…4 s delay between CBFV(t) and ABP(t) signals. Examples in Fig. 3c and d show cases of disturbed cerebral blood flow autoregulation with baroreflex failure for patients with unfavorable outcomes: DCI and severe disability (Fig. 3c) and fatal outcome (Fig. 3d).

**Fig. 4:**
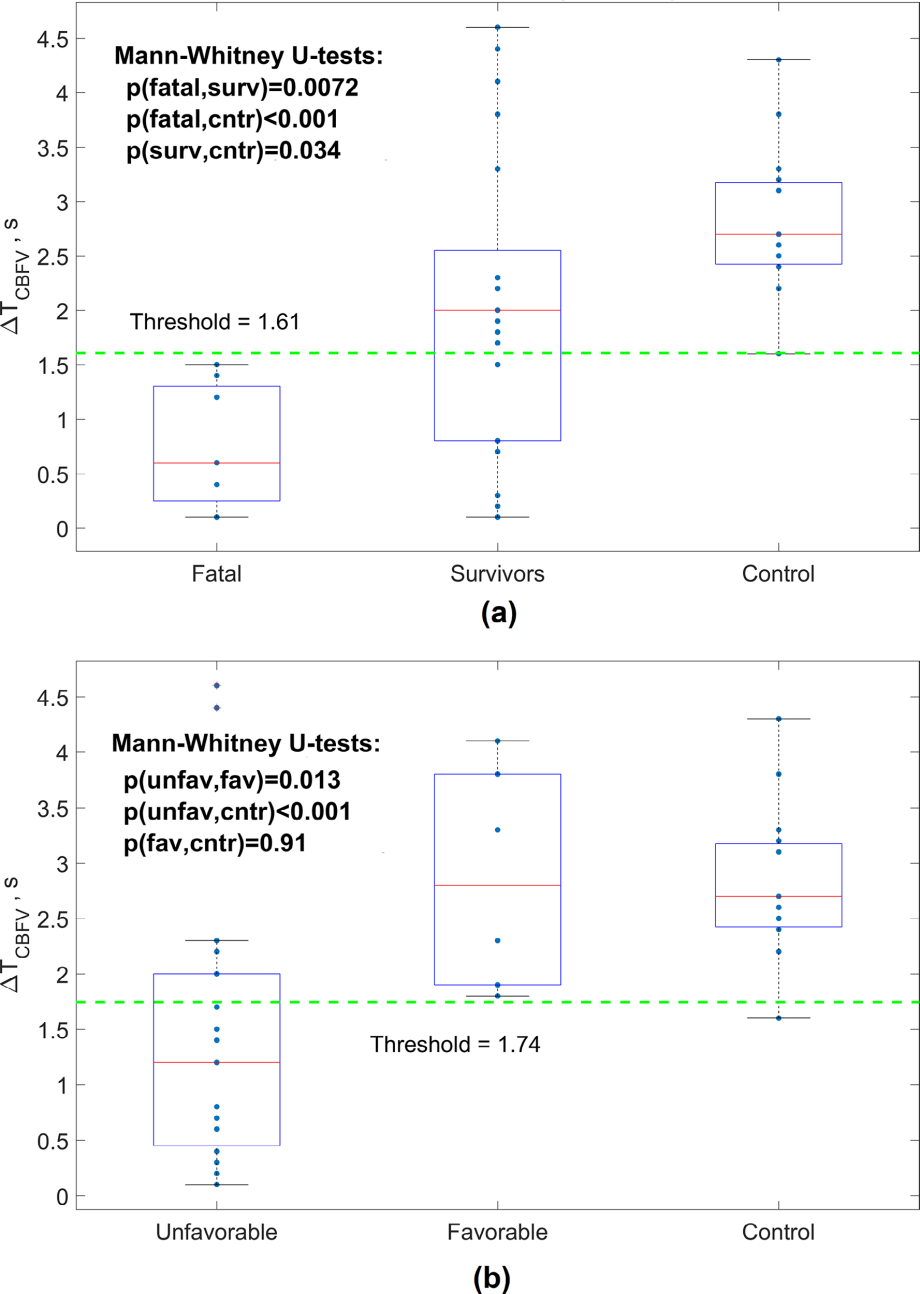
Distribution of delay time between ABP(t) and CBFV(t) during deflation cycle (∆TCBFV) identified for SAH patients with fatal outcome (GOS 1), survivors (GOS > = 2) and healthy control group (**a**), and for SAH patients with unfavorable (GOS < = 3) and favorable (GOS > 3) outcomes and the healthy control group (**b**). Dashed lines show the threshold values that statistically significant separate fatal and survivors (**a**) and unfavorable and favorable patients groups (**b**)

Statistically significant differences in CA-related delay ∆T_CBFV_ were found among the fatal outcome patient group (∆T_CBFV_ = 0.74 ± 0.58 s), survival group (∆T_CBFV_ = 2.05 ± 1.35 s) and healthy control group (∆T_CBFV_ = 2.76 ± 0.75 s) (Fig. 4a and b). Statistically significant differences in delay ∆T_CBFV_ were also found between the unfavorable outcome group (∆T_CBFV_ = 1.37 ± 1.23 s) and the favorable outcome group (∆T_CBFV_ = 2.86 ± 0.99 s). ∆T_CBFV_ for the healthy control group also significantly differed from that of patients with unfavorable outcomes (*p* < 0.001) but not from that of patients with favorable outcomes (Fig. 4b).

**Fig. 5:**
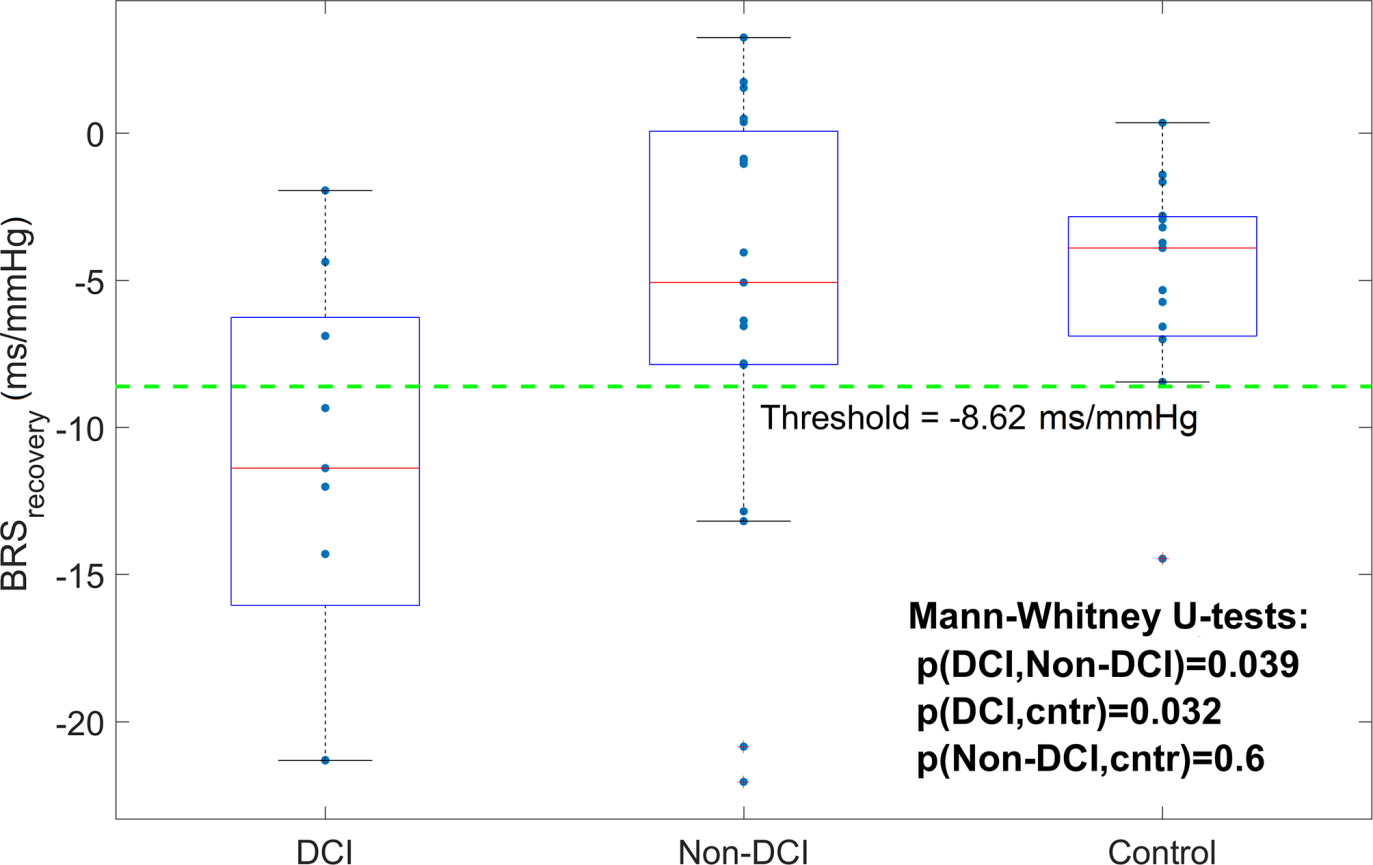
Distribution of BRS-related factor (∆T_IBI_/∆ABP_recovery_) calculated at the recovery phase after the ABP drop among SAH patients with DCI and non-DCI and the healthy control group. Dashed line shows the threshold value that statistically significant separate fatal and DCI and non-DCI patients groups

The analyzed CA-related parameters, either Mx or ∆T_CBFV_, showed significant associations with CV and DCI occurrence. The BRS-related index at the boot deflation cycle (BRS_recovery_) showed statistically significant differences between the patient group who experienced DCI and those without DCI (*p* = 0.039) (Fig. 5). However, BRS_recovery_ and BRS_drop_ did not show statistically significant associations with patient outcomes or CV patient groups.

## Discussion

In this study, we investigated the impact of CA impairments detected in the early period on SAH patient outcomes by performing snapshot TCD examinations with artificial ABP manipulations. We chose the delay time between ABP(t) and CBFV(t) during induced ABP changes (∆T_CBFV_) as a CA-related predictor of SAH patient outcome. Higher ∆T_CBFV_ values (2.86 ± 0.99 s) measured in the early period of the clinical course (2–3 days after SAH) were associated with favorable SAH patient outcomes, whereas lower values (∆T_CBFV_ < 1.7 s) were observed for patients with unfavorable outcomes. We found no significant associations between ∆T_CBFV_ and the occurrence of DCI or CV. In our study, not only DCI but also other complications, such as cerebral edema, hydrocephalus, sepsis, and pneumonia, were leading causes of unfavorable outcomes after SAH. The total number of unfavorable cases was 78%, compared to 29% of DCI cases.

A moderate correlation between ∆T_CBFV_ and the noninvasive Mx index was found in the study population (*r*=-0.461, *p* = 0.012), confirming that both measures show an indirect association with CA status. However, the Mx index estimated in the early period of SAH patients’ treatment during the examination sessions with inflatable boots did not show a statistically significant association with patients’ outcome or DCI/CV events.

In this study, we applied a pressotherapy device capable of generating safe controlled changes in ABP instead of thigh cuff release methods, which are often used for dynamic CA assessment. The main disadvantage of the thigh cuff technique is the risk of secondary brain injuries in ischemic stroke patients when ABP drops by up to 30 mmHg during a short deflation cycle. Furthermore, the rapid decrease in ABP may induce additional responses due to baroreflex triggering or changes in arterial partial pressure of carbon dioxide (pCO2), which may independently affect blood flow and ABP changes [35]. In our study, we used a pressure-based therapeutic massage device (leg recovery device (Reboots), Germany) with a controlled boot inflation pressure (not exceeding 200 mmHg) and ABP drop amplitude (not exceeding 10…15 mmHg per 10 s deflation cycle). Similar devices are used in most ICUs to prevent deep vein thrombosis, venous thromboembolism, as well as to improve blood circulation for long-term patient treatment.

To explore the impact of baroreflex on cerebral blood flow regulation during artificially induced ABP changes, we also plotted averaged responses of HR, ABP and CBFV during the boot deflation cycle (Fig. 3). For healthy volunteers, an increase in HR is observed due to triggered baroreflex after a sudden fall in ABP, resulting in a regulation of CBFV, thus causing a leftward shift of the CBFV nadir peak with respect to the ABP nadir (Fig. 3a).

However, for SAH patients, the symptoms of baroreflex failure [36] were observed in almost all examination cases; the impact of cardiovascular response after the fall in ABP on CBFV regulation was not observed in either favorable or unfavorable outcome groups of SAH patients, despite the autoregulatory responses in CBFV. This allows us to hypothesize that a leftward shift in CBFV with respect to ABP during inflation is achieved through autoregulatory interactions of various neurogenic, hemodynamic, autoregulatory, and metabolic factors [37], whereas baroreflex is not the only factor affecting the autoregulatory blood flow response during the initial phase of the ABP drop [37, 38]. A decrease in ABP triggers an integrated physiological response that not only involves arterial baroreflex-mediated changes in HR and peripheral vasculature resistance but also induces a cerebrovascular autoregulatory response [36, 37].

Analysis of BRS-related factors did not reveal a significant association with the outcome of patients with SAH. However, a statistically significant association was observed between the baroreflex-related index BRS_recover_ (calculated as ∆T_IBI_/∆ABP_recover_ ratio during the recovery phase after ABP drop) and DCI events (Fig. 5; Table 3). Examples of averaged HR and CBFV responses for SAH patients who experienced DCI are shown in Fig. 3c in comparison with other SAH patients without DCI (Fig. 3b and d) and healthy volunteers (Fig. 3a).

**Table 3:** Patient demographics and clinical examination data (patients with and without delayed cerebral ischemia and healthy control groups)

	DCI group	Non-DCI group	Healthy control	p (DCI, Non-DCI)	p (DCI, Cont.)	p (Non-DCI, Cont.)
Number	8	21	15	-	-	-
Age, years median [IQR]	68 [65–77]	65 [53–75]	37 [33–40]	0.59	< 0.001*	< 0.001*
Sex, M/F	4/4	12/9	12/3	0.76	-	-
GCS, IQR	[8–12]	[5–11]	-	0.35	-	-
CV, n	8	2	-	< 0.001*	-	-
∆T_CBFV_, s mean (std)	1.57 (0.80)	2.05 (1.35)	2.76 (0.72)	0.73	0.002*	0.012*
Mx mean (std)	0.32 (0.16)	0.34 (0.23)	0.33 (0.08)	0.79	0.94	0.72
BRS_drop,_ ms/mmH mean (std)	1.14 (10.1)	-3.78 (7.34)	9.65 (6.86)	0.13	0.049*	< 0.001*
BRS _recovery,_ ms/mmHg mean (std)	-11.4 (6.79)	-5.65 (7.26)	-5.67 (4.94)	0.039*	0.032*	0.6

GCS is the Glasgow coma scale, CV is the cerebral vasospasm, DCI is the delayed cerebral ischemia, ∆T_CBFV_ is the time difference between cerebral blood flow velocity and arterial blood pressure, Mx is the mean flow autoregulation index, BRS_drop_ and BRS_recovery_ are the baroreflex sensitivity indices calculated as ratios between changes in interbeat interval and changes in arterial blood pressure (∆T_IBI_/∆ABP) during the boot deflation cycle at falling and recovery moments, IQR is the interquartile range, std is the standard deviation, and Cont. is the control group. The asterisk (*) indicates statistical significance (*p* < 0.05)

The impact of BRS on SAH patients’ clinical course has been analyzed in several studies [30–33]. It was shown that an inverse compensatory correlation between BRS and CA is observed for SAH patients who avoided CV occurrence, whereas this relationship was lost in patients who experienced CV, both before and during vasospasm [30]. Decreased BRS within the first five days following SAH was found to be associated with regional cerebral deoxygenation [31] and consequently with poor outcomes [32, 33].

### Limitations

The main limitation is the relatively small number of patients included in the study, which might affect the reliability of statistically significant factors. The obtained relationship between the CA-related biomarkers used in the study and patient outcomes might be affected by secondary complications following SAH, not DCI alone [39]. Additional factors - age and Glasgow coma scale (GCS) - also showed statistically significant associations with patient outcomes; therefore, a larger number of participants is necessary for the further development of more accurate multivariate outcome prediction models.

We limited our study to BRS and CA-related factors evaluated from short 20-minute examination cycles performed 2–3 days after SAH, while CV occurred in 4–9 days. More frequent examinations of SAH patient parameters would be more rational during the overall clinical course. However, CA assessment by using TCD and pressotherapy devices became complicated in semiconscious and disorientated patients after they awakened from surgery sedation. Moreover, TCD examinations were performed only on one intact side of the head after brain aneurysm surgery.

Some patients were sedated during examinations. Some patients in our study received administration of vasopressors at individually adjusted dosages to regulate ABP. Although, applied pharmaceutical treatment by anesthetics (mainly propofol) and vasopressors can affect both CA and BRS test results by blocking the cardiovascular reflex response [40] or affecting cerebrovascular reactivity [41, 42], however, recent study showed that propofol did not produce a significant alteration in the average heart rate, which serves as the basis for measuring baroreflex sensitivity [43].

Additional limitations are related to patient outcome assessment and CV/DCI diagnosis. Patients’ outcome data were available only at hospital discharge. CV and DCI were diagnosed according to clinical symptoms or CT/CTA scan examinations (data obtained from the hospital database). Therefore, CVs are clearly seen only in large cerebral arteries, while spasm detection in smaller arteries (with diameters < 3 mm) can be missed.

## Conclusions

The proposed methodology based on TCD and inflatable leg recovery boots application allows safe assessment of CA status within a short period of time − 20 min. CA-related biomarker—delay between CBFV(t) and ABP(t) during the boot deflation cycle—measured during snapshot TCD examination in the early prevasospasm period showed a significant association with SAH patient outcome but not with CV and DCI. The BRS-related ratio between changes in the heartbeat period (∆T_IBI_) and ABP at the boot deflation cycle assessed in the early prevasospasm phase was significantly associated with later occurrence of DCI. Not only DCI but also other complications, such as cerebral edema, hydrocephalus, sepsis, and pneumonia may worsen clinical outcomes after SAH.

## Data Availability

The datasets used and analyzed during the current study are available from the corresponding author on reasonable request.

## Abbreviations

ABP: arterial blood pressure
BRS: baroreflex sensitivity
CA: cerebral blood flow autoregulation
CBFV: cerebral blood flow velocity
Cont.: control group
CPP: cerebral perfusion pressure
CT: computed tomography
CTA: computed tomography angiography
CV: cerebral vasospasm
DCI: delayed cerebral ischemia
GOS: Glasgow outcome scale
GCS: Glasgow coma scale
ICU: Intensive care unit
IQR: interquartile range
MCA: middle cerebral artery
Mx: mean flow autoregulation index
pCO2: arterial partial pressure of carbon dioxide
PS: phase shift
PRx: Pressure reactivity index
SAH: subarachnoid hemorrhage
Surv.: survivor group
std: standard deviation
TCD: transcranial Doppler
